# Effects of Obesity on Adiponectin System Skin Expression in Dogs: A Comparative Study

**DOI:** 10.3390/ani11082308

**Published:** 2021-08-05

**Authors:** Cecilia Dall’Aglio, Margherita Maranesi, Antonio Di Loria, Diego Piantedosi, Paolo Ciaramella, Maria Chiara Alterisio, Elvio Lepri, Francesca Mercati

**Affiliations:** 1 Department of Veterinary Medicine, University of Perugia, Via San Costanzo 4, 06126 Perugia, Italy; cecilia.dallaglio@unipg.it (C.D.); elvio.lepri@unipg.it (E.L.); francesca.mercati@unipg.it (F.M.); 2 Department of Veterinary Medicine and Animal Productions, University Federico II of Napoli, Via F. Delpino 1, 080137 Napoli, Italy; adiloria@unina.it (A.D.L.); diego.piantedosi@unina.it (D.P.); paociara@unina.it (P.C.); mariachiara.alterisio@unina.it (M.C.A.)

**Keywords:** ADIPOQ, ADIPOR1, ADIPOR2, RT-qPCR, immunohistochemistry, integumentary system, dog

## Abstract

**Simple Summary:**

Adipokines are biologically active molecules with hormonal action, produced mainly by white adipose tissue and related to the individual’s nutritional status. Adiponectin with its receptors (ADIPOR1, ADIPOR2) intervenes in the control of energy metabolism, as well as in the regulation of peripheral tissue functions. Adiponectin has a primary role in the skin in both physiological and pathological conditions, in addition, this molecule is greatly affected by nutritional status, and its serum level is lowered in the obese. In this work, the adiponectin system was evaluated in the skin of obese dogs along with adiponectin serum levels. Results were compared to normal weight dogs to evidence modifications in the obesity condition. Obesity is a widespread phenomenon in dogs, with a growing trend, as well, in humans; this condition may interfere with the local functionality of tissues, including the skin. The evaluation performed evidenced that adiponectin and ADIPOR2 skin expression is negatively correlated with the serum adiponectin level and accordingly with obesity. These findings evidence that the adiponectin system changes in the skin of obese dogs; this study also explores the role of adipokines in skin biology.

**Abstract:**

Obesity is an important health issue in dogs since it influences a plethora of associated pathologies, including dermatological disorders. Considering the scarcity of information in pets, this work aimed to evaluate the localization and expression of adiponectin (ADIPOQ) and its two receptors (ADIPOR1 and ADIPOR2) in the skin of 10 obese dogs, compared with serum ADIPOQ level. Through immunohistochemistry, ADIPOQ and ADIPOR2 were observed in the adipose tissue, sweat and sebaceous glands, endothelium, and some connective cells. Both receptors were observed in the epidermis and the hair follicles, other than in the sweat and sebaceous glands. Real-time PCR evidenced that the *ADIPOQ* and *ADIPOR2* transcripts were expressed 5.4-fold (*p* < 0.01) and 2.3-fold less (*p* < 0.01), respectively, in obese than in normal weight dogs, while *ADIPOR1* expression did not change. Obese dogs showed lower serum ADIPOQ levels than the normal weight group. Accordingly, ADIPOQ and ADIPOR2 expression in the skin appear negatively correlated with obesity in the same way as the serum ADIPOQ level. These findings evidence that ADIPOQ system changes in the skin of obese dogs and suggest that the ADIPOQ effect on the skin is at least in part regulated by the reduced expression of ADIPOR2.

## 1. Introduction

Obesity is a critical health issue in dogs, and surveys assessing obesity in the canine population suggest there is a high global incidence of this phenomenon [1]. Obesity can s be defined as excessive storage of adipose tissue and it occurs when animals are in a positive energy balance for an extended period of time. A dog can be considered obese when its body weight exceeds the optimum weight for the body size by at least 15% [2]. Canine obesity is related to lifestyle and is commonly observed that overweight people are more likely to have overweight dogs [3]. However, obesity in dogs is an alteration of the dog’s energy metabolism; it cannot simply be attributed to the owner’s inability to manage its pet’s food. Research is suggesting that obesity is a complex physiological issue, with genetic and endocrine influences that predispose some individuals to become obese [4,5,6]. Obesity also represents a significant welfare problem for pet dogs, resulting in adverse effects on health and longevity [7]. Obese dogs die sooner and have a higher incidence of several clinical conditions, including dermatological disorders, when compared to non-obese dogs.

Given the growing number of obese dogs and clinical implications, the study on the mechanisms and molecules involved in obesity is of particular interest. Adipose tissue is an active endocrine organ and releases a variety of bioactive molecules, known as adipokines, which affect whole-body homeostasis. Adipokine levels may vary in the obese according to adipose tissue accumulation. In addition to adipose tissue, several peripheral tissues secrete adipokines [8,9,10,11,12,13], where they perform local actions that can be affected by obesity [14]. Adiponectin (ADIPOQ) is the most abundant gene product in adipose tissue and accounts for 0.01% of total plasma protein [15]. The plasma concentration of ADIPOQ decreases in conditions of obesity and increases with weight loss [15,16]. Arita et al. [15] suggested that dysregulation of ADIPOQ may be relevant to obesity-linked disorders. ADIPOQ is a 28-kDa protein hormone composed of 244 amino acids encoded by the ADIPOQ gene [17]. It is mainly produced by adipose tissue; however, it is a pleiotropic hormone and its expression, along with its receptors, was described in several peripheral tissues [18], including the skin [8,10]. The most important biological role of ADIPOQ is the insulin-sensitizing action. The anti-inflammatory action is also known. This function is performed by the hormone in various pathological conditions, including non-alcoholic fatty liver disease (NAFLD), type 2 diabetes, and cardiovascular disease [19]. The main targets of the anti-inflammatory action of ADIPOQ are macrophages [20].

ADIPOQ acts by binding to its receptors: ADIPOR1 and ADIPOR2. ADIPOR1 is almost ubiquitous and particularly abundant in skeletal muscle and liver, while ADIPOR2 is mainly expressed in the liver. Structurally, ADIPOR1 and ADIPOR2 possess seven transmembrane domains, an extracellular carboxy-terminal domain, and an intracellular amino-terminal domain [21].

ADIPOQ has important roles in skin physiology since it is involved in several function, such as proliferation and migration of keratinocytes and sebocytes [22,23], regulation of re-epithelialization processes, production of sebaceous lipids [23], and stimulation of hair growth by promoting the proliferation of keratinocytes of the cellular matrix [24]. ADIPOQ plays a role in wound repair by promoting the migration and proliferation of human keratinocytes and endothelial cells [22,25] and, accordingly, angiogenesis and re-epithelialization through the ERK pathway. ADIPOQ levels influence skin diseases, such as psoriasis and acne vulgaris [26,27,28]. 

ADIPOQ promotes the proliferation of dermal fibroblasts and the upregulation of type I collagen [29] and hyaluronic acid (HA) by increasing the level of hyaluronic acid synthetase 2 (HAS2) in human skin fibroblasts [30]. HAS2 is the largest producer of HA, which in turn maintains skin hydration, facilitates the transport of ions and nutrients, and promotes wound repair [30]. Since this molecule is greatly affected by nutritional conditions and its serum level is lowered in the obese [16], it has been hypothesized that skin hydration, thickness, and elasticity are compromised in rats fed high-fat diets because of the low levels of ADIPOQ, which do not effectively stimulate collagen and HA production [30]. However, previous works evaluated variation of serum ADIPOQ level, while the molecule system was not considered at the skin site in the obese. 

Considering the primary role that ADIPOQ has in the skin and the scarcity of investigations in the veterinary field, the purpose of this work was to evaluate ADIPOQ system in the skin of obese dogs via immunohistochemistry and real-time PCR, and to compare its local modifications with serum ADIPOQ level.

## 2. Materials and Methods

### 2.1. Recruitment of the Animals

Twenty mixed breed, medium-sized dogs were recruited; the animals were the same as in a previously published paper, to which we refer to [14]. All animals were healthy as verified by clinical, hematological, and biochemical examination, and devoid of pre-existing pathological conditions. No animals were pregnant or nursing. Recruited animals were fed a homemade chicken protein-based diet. They were divided into two groups: 10 obese dogs (obese group, 6 males and 4 females) and 10 normal weight dogs (normal weight group, 6 males and 4 females). The mean age was 5.9 ± 1.2 years in the obese group and 5.3 ± 1.3 years in the normal weight group. Body condition score (BCS) was evaluated according to the nine-point BCS system [31]. Experimental procedures were approved by the Ethical Animal Care and Use Committee (n. PG/2017/0099607) of the University of Naples “Federico II”, Naples.

### 2.2. Complete Blood Count (CBC), Serum Biochemistry and Adiponectin Assay 

A blood sample was obtained from each dog after jugular venipuncture using EDTA tubes and tubs with serum separator. CBCs were performed using a semi-automatic cell counter (Genius S, SEAC Radom Group). After centrifugation at 327 *g* for 10 min, a semi-automatic chemical chemistry analyzer (OLOT, Spinreact) was used to analyze serum concentrations or activities of glucose, blood urea nitrogen (BUN), creatinine, triglycerides, total cholesterol, alanine aminotransferase (ALT), alkaline phosphate (ALP), total bilirubin (T-Bil), gamma glutamyl transferase (GGT), albumin, and total serum proteins; serum proteins electrophoresis was also performed. Serum ADIPOQ concentrations were measured in triplicate using commercial canine ELISA kits (Cloud-Clone Corp., Houston, TX, USA).

### 2.3. Sample Collection: Skin Biopsies

Skin biopsies (1 cm^2^) were collected from the ventral region during surgical neutering. For histochemical procedures, biopsies were quickly fixed in 10% neutral buffered formalin solution in phosphate buffered saline (PBS 0.1 M, pH 7.4) and processed until they were embedded in paraffin wax [12]. Sections with a thickness of 5-µm were mounted onto poly-L-lysine coated glass slides and stained with hematoxylin–eosin and Giemsa, to exclude the presence of skin lesions and to evaluate the number of mast cells respectively [14]. Mast cells were blindly evaluated, counting their numbers on five contiguous high-power fields (magnification 400× with a field number of the ocular of 22) for each section. The total number of mast cells was recorded and the mean value for the 2 operators was assessed [32]. For the molecular biology test, skin biopsies were immediately frozen in liquid nitrogen and stored at −80 °C until it was time to measure the transcript expression.

### 2.4. Immunohistochemistry

Immunohistochemistry was conducted as follows [33]. Rehydrated skin sections were dipped for 10 min in 3% H2O2 to reduce endogenous peroxidase activity, incubated with HistoReveal (Abcam, Cambridge, UK) for 5 min to perform antigen retrieval and, then, incubated for 30 min with normal serum (1:10 with PBS; Vector Laboratories, Burlingame, CA, USA). Incubation with the primary antibodies was performed overnight at room temperature with the following sera: rabbit anti-canine ADIPOQ Polyclonal Antibody (1:50 with PBS; MBS2028428, MyBioSource, CA, USA), rabbit polyclonal anti ADIPOR1 (1:100 with PBS; LS-C151518; LifeSpan BioSciences, WA, USA) and ADIPOR2 (1:100 with PBS; ARP60819_P050, Aviva Systems Biology Corporation, CA, USA). Starting from the normal serum, all steps were performed incubating the slides in a humid chamber. On the second day, the sections were incubated for 30 min with horse anti-rabbit biotin-conjugated secondary antibodies (1:200 with PBS; BA-1100; Vector Laboratories, Burlingame, CA, USA). The immune complexes were visualized using an avidin-biotin system (Vectastain ABC kit; Vector Laboratories, Burlingame, CA, USA) and diaminobenzidine (DAB) as chromogen (Vector Laboratories), considering as positive the cells and structures where immunostaining was visible. The nuclei were counterstained with Mayer’s Hematoxylin. Sections of visceral adipose tissue were used as positive control [34,35]. Negative controls were made by incubating sections with normal rabbit IgG. For each slide, two independent investigators assessed the localization of the labeling. All histological sections were observed at 200×, 400×, and 1000× magnification under a photomicroscope (Nikon Eclipse E800, Nikon Corp., Tokyo, Japan) connected to a digital camera (Nikon DXM 1200 digital camera, Nikon Corp.). 

### 2.5. RNA Extraction and Real-Time PCR

Total RNA was extracted from the skin biopsies of 10 dogs from each experimental group, as previously described [14]. Five µg of total RNA was reverse transcribed in 20 µL of iScript cDNA using random hexamer according to the protocol provided by the manufacturer (Bio-Rad Laboratories, Milan, Italy). Genomic DNA contamination was checked by developing a PCR without reverse transcriptase. Serial experiments were carried out to optimize the quantitative reaction, efficiency, and Ct values. The optimal 25 µL PCR reaction volume contained 12.5 µL of iQ SYBR Green SuperMix (Bio-Rad Laboratories), 1 µL forward and reverse primers (stock concentration of 10 µM), and water to 25 µL. The primers used are listed in Table 1.

**Figure 1:**
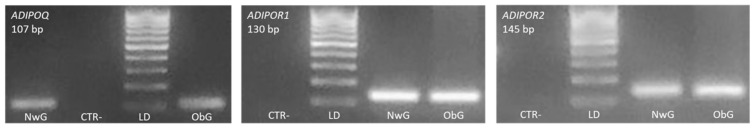
Representative photographs of typical 2% agarose ethidium bromide stained gels. The presence of the expected bp products yielded after RT-qPCR using primers for target *ADIPOQ* and its receptors (*ADIPOR1* and *ADIPOR2*) are shown. Lane LD is the kilobase DNA marker, CTR- is the negative control, lane NwG (normal weight group) and ObG (obese group) identify two skin samples belonging to the two experimental groups.

All reagents were mixed as a master mix and distributed into a 96-well PCR plate before adding 2 µL of cDNA (10-fold diluted with water). For every PCR run, reaction controls without template and reverse transcriptase in RT were included as negative controls to ensure that RNA was free of genomic DNA contamination. The amplification fidelity of samples was also verified by agarose gel electrophoresis for two animal subjects belonging to the different groups (Figure 1). The images of gels were acquired by using a Kodak DC290 digital camera. 

RT-qPCR was performed on an iCycler iQ (Bio-Rad Laboratories) with an initial incubation at 95C for 1.5 min, followed by 40 cycles at 95C for 15 s, 53C for 30 s, during which fluorescence data were collected. The threshold cycle (Ct value) was automatically computed for each trace. PCR products were purified and sequenced by QIAquick PCR Purification Kit, according to the manufacturer’s protocols (Qiagen Inc., Milan, Italy).

The beta-actin Ct housekeeping gene (*ACTB*) was determined in order to normalize sample variations in the amount of starting cDNA.

Standard curves were generated by plotting the threshold value (Ct) against the log cDNA standard dilution (1/10 dilution) in nuclease-free water. The slope of these graphs was used to determine the reaction efficiency. Sample mRNA quantification was evaluated using iCycler system software, while mRNA gene expression was quantified using the 2^−ΔΔ^Ct method [9,36]. The melting curve analysis was carried out, immediately after the PCR end cycle, to determine the specificity of each primer set. A melt-curve protocol was performed by repeating 80 heating cycles for 10 s, from 55C with 0.5C increments, during which fluorescence data were collected.

### 2.6. Statistical Analysis

Data obtained by hematochemical evaluation were assessed by the Shapiro–Wilk test, to test for normality, and where necessary, logarithmic transformation was performed to compare the normal weight and obese groups using Student’s *t* test.

Data on gene expression and protein were examined by ANOVA followed by Student–Newman–Keuls t-test. All values are means ± SD for each dog groups; differences are considered significant at *p* < 0.05 [37].

## 3. Results 

### 3.1. Animal Body Weight and Body Condition Score

The BW and BCS recorded in the obese group were 35.0 ± 9.2 kg and 7.9 ± 0.5, respectively; compared with 18.7 ± 3.4 kg and 5 ± 0, respectively for the normal weight group. The two groups showed a significant difference in BW (*p* < 0.01), while no significant difference for BW was seen with respect to sex. 

### 3.2. Serum Biochemical Profile

Table 2 shows the results of serum biochemical profile. Briefly a significant difference between the two groups occurred for α-globulin fraction (*p* < 0.01), GGT and T-Bil (*p* < 0.05), while the obese group showed lower levels of ADIPOQ (183.05 ± 83.2 pg/mL) than the normal weight group (*p* < 0.0001).

### 3.3. Histology and Immunohistochemistry

Histologically, the skin biopsies did not show any lesions, and no differences in the total number of mast cells were present between groups.

The immunohistochemical investigation revealed the presence of ADIPOQ, ADIPOR1, and ADIPOR2 in the skin of the dogs, in both obese and normal weight subjects.

**Figure 2:**
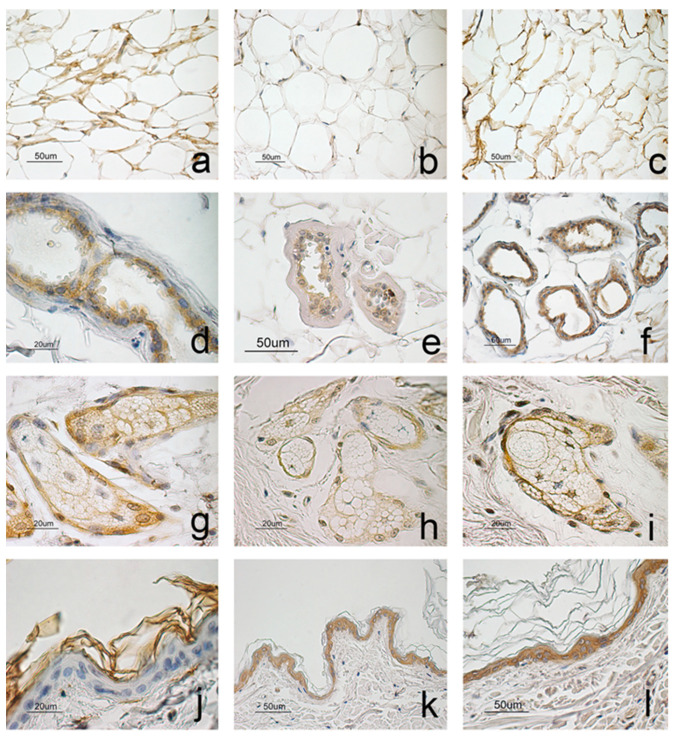
Immunostaining for ADIPOQ (**a**,**d**,**g**,**j**), ADIPOR1 (**b**,**e**,**h**,**k**), and ADIPOR2 (**c**,**f**,**i**,**l**). ADIPOQ (**a**) and ADIPOR2 (**c**) stained adipose tissue located among hair follicles that instead appeared negative to ADIPOR1 (**b**). In the sebaceous and sweat glands, positivity appeared stronger to ADIPQ (**d**,**g**) and ADIPOR2 (**f**,**i**) respect to ADIPOR1 (**e**,**h**). ADIPOQ (**j**) did not stain the epidermis while both receptors were observed in all epidermal cell layers (**k**,**l**).

**Figure 3:**
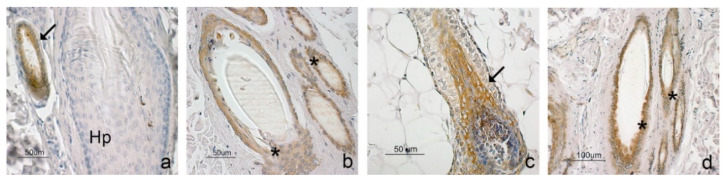
Immunostaining for ADIPOQ (**a**), ADIPOR1 (**b**,**c**), and ADIPOR2 (**d**) in primary and secondary hair follicles. (**a**) An ADIPQ-negative hair follicle (Hp) is close to an ADIPOQ-positive sebaceous gland (arrow). (**b**) A group of hair follicles positive to ADIPOR1. Staining is localized in the outer root sheath cells (asterisk). (**c**) A hair follicle bulb where the ADIPOR1-positive inner root sheath (arrow) is shown. (**d**) A group of hair follicles positive to ADIPOR2. Positivity can be observed in the outer root sheath cells (asterisk).

ADIPOQ was mainly observed in the adipose tissue extending among follicular clusters (Figure 2a), in the sweat and sebaceous glands (Figure 2d,g), in the endothelium and some connective cells. Epidermis (Figure 2j) and hair follicles (Figure 3a) did not stain positively.

ADIPOR1 and ADIPOR2 showed a wider immunostaining than the ligand. Both receptors were observed in all cell layers of the epidermis (Figure 2k,l) and in the outer root sheath of the hair follicles at the level of the isthmus (Figure 3b,d); besides, ADIPOR1 was also observed in the inner root sheath, mainly in the bulbar region (Figure 3c). Immunopositivity to both receptors was also observed in the sweat and sebaceous glands (Figure 2e,f,h,i); however, the staining appeared weak concerning ADIPOR1, while it was intense in the case of ADIPOR2. Finally, positivity for ADIPOR2 was observed in the adipose tissue (Figure 2c), in some connective cells and the capillary endothelium.

### 3.4. ADIPOQ, ADIPOR1, and ADIPOR2 Gene Expression by Real-Time PCR

The *ADIPOQ* and its cognate receptors transcripts (*ADIPOR1*, *ADIPOR2*) were evidenced in all dog skin specimens. The *ADIPOQ* and *ADIPOR2* transcripts were expressed 5.4-fold (*p* < 0.01) and 2.3-fold less (*p* < 0.01), respectively, in obese subjects than in normal weight subjects (Table 3). There were no differences in *ADIPOR1* mRNA levels between the two groups (Table 3).

## 4. Discussion

This work describes the evaluation of ADIPOQ and its receptors on the skin of obese dogs; our study aimed to detect the presence of ADIPOQ system in the skin and its variation in the obesity condition. To our knowledge, this is the first description of a downregulation of ADIPOQ and ADIPOR2 in the skin of obese dogs; thus, showing a possible role of adiponectin in this serious health problem. This study expands the knowledge on the role of adipokines in the skin, starting from previous evaluations in dogs [39], with respect to obesity [14].

Regarding metabolic status, obese dogs showed increased BCS, elevated serum α-globulin fraction, and reduced serum ADIPOQ levels than normal weight animals. The higher levels of serum α-globulin fraction in the obese group can be due to an increase in specific acute phase proteins, suggesting a potential inflammatory state, frequently observed in obese patients [40]. The significant differences observed in GGT and T-Bil concentrations between normal weight and obese dogs may be closely related to cholestasis and biliary retention, findings that are described in previous studies on canine obesity [41,42]. The pathophysiological mechanism may be similar to that observed in humans, in which obesity led to development of biliary diseases, because of excessive hepatic secretion of cholesterol, following by supersaturation of bile, increasing gallbladder volume and impairment of the gallbladder contraction [43]. No increase of cholesterol and triglycerides were detected in the obese group, probably due to the strict protein diet administrated to all dogs enrolled. We should note that, in human obese patients, the high-protein diet also commonly used for weight loss is able to control serum triglyceride levels and cholesterol [44].

The obese group showed a low level of serum ADIPOQ in accordance with results observed in previous studies on cohorts of dogs with naturally occurring obesity [45,46]. These results confirm the “counterbalance role” of this adipokine, with respect to leptin in energy metabolism homeostasis, validating its anti-inflammatory, pro-apoptotic, and anti-proliferative effects in obesity conditions [45,47].

In dog skin, ADIPOQ immunostaining appeared in the adipose tissue, in the sebaceous and sweat glands, in vessel endothelium, and some connective tissue cells. The results obtained are partially different from Brèment et al. [10], who studied ADIPOQ localization in the skin of a dog, describing the molecule at the level of the epidermis, follicular wall, sebaceous and sweat glands, and vascular endothelium. ADIPOQ is expressed and secreted by subcutaneous adipose tissue other than visceral adipose tissue [8,34,48]. Moreover, Akazawa et al. [8] proposed sebaceous gland cells as an ADIPOQ source of the skin, together with subcutaneous fat, but they cannot detect ADIPOQ mRNA in keratinocytes nor dermal fibroblast. ADIPOQ localization in dog skin suggests that the molecule produced by adipose tissue, extending among hair follicles, acts on the skin structures expressing the receptors with a paracrine type mechanism. In addition, an autocrine mechanism can also be supposed for sebaceous and sweat glands. 

ADIPOR1 and ADIPOR2 were observed in the epidermis, hair follicles, sweat, and sebaceous glands, vascular endothelium, and some connective tissue cells. Receptor immunostaining revealed the structures and cells of the dog skin reactive to the action of the molecule under investigation.

In regard to the epidermis, an intense positivity for both ADIPOR1 and ADIPOR2 was observed in all cell layers in analogy with humans [24]. The expression of the receptors was also described on cell cultures of keratinocytes [8,22], where ADIPOQ stimulates cell proliferation and migration mediated by receptors through the ERK signaling pathway [22]. The epidermis, therefore, seems to be sensitive to the action of ADIPOQ, which appears to be involved in the regulation of the tissue turnover, as well as in the regeneration processes following skin wounds. In this latter situation, the action of the molecule is also directed toward the vascular endothelium [25,49] and skin fibroblasts [29], where the receptors are expressed, stimulating vascularization and granulation tissue formation. ADIPOQ precisely promotes the synthesis of hyaluronic acid (HA) by increasing the level of HA synthetase 2 in human skin fibroblasts [29,30]. The positivity of ADIPOR1 and ADIPOR2, which was observed in the epidermis, in the vascular endothelium, and connective tissue cells allows us to hypothesize that, even in the dog’s skin, ADIPOQ carries out a similar role to that described in humans. 

The RT-qPCR analysis revealed significant differences on the *ADIPOQ* and *ADIPOR2* transcript expression between the obese and the normal weight group, while no significant differences were observed with regard to the *ADIPOR1*. The *ADIPOQ* transcript was found expressed 5.4 times less in the obese group than the normal weight group, while the *ADIPOR2* transcript was 2.3 times lower in the obese group. This outcome demonstrates a negative correlation between the ADIPOQ and ADIPOR2 levels expressed in the skin and in the body fat mass that is the obesity condition. Obese dogs used in this study had a reduced plasma ADIPOQ concentration compared to the normal weight subjects [46]. Therefore, the concentration of cutaneous ADIPOQ and ADIPOR2 appears positively correlated with the molecule plasma levels. These results suggest that obese dogs could present an alteration of the skin functions related to ADIPOQ, such as the stimulation of the cell proliferation and migration. The ADIPOQ and ADIPOR2 lower expression could cause a reduced synthesis of HA and, consequently, expose the skin to less hydration, dysfunctions, and an inefficient wound healing process in the obese.

In addition to the epidermis, immunohistochemical positivity for both receptors was also observed in the hair follicles, where small differences with humans were observed [24] (Won et al., 2012). The ADIPOR1 and ADIPOR2 positive cells were mainly observed in the outer root sheath of the isthmic region. Furthermore, ADIPOR1 was observed in the internal root sheath at the level of the bulbar region of the anagen phase follicles. Unlike humans, no positivity was observed in the dermal papilla. The outer and inner root sheaths are composed of epithelial cells that form the follicular wall and are primarily responsible for the growth and regression phases of the hair follicle [50]. The outer root sheath contains a population of stem cells [51,52], whose telogen periodic activation allows regeneration of the bulbar follicular portion [53]. The cells of the inner root sheath accompany the growing hair, favoring its development, stability, and anchoring to the follicular structure [54,55,56]. ADIPOQ was proposed as a hair growth stimulator since it promotes hair growth in organ cultures in a dose-dependent manner [24] and cell proliferation of the pilosebaceous units of the healing tissue [57]. Accordingly, the reduced expression of ADIPOQ and ADIPOR2 in obese dogs could promote the onset of follicular dysfunctions, including an alteration and delay in the follicular growth phase that could result in an altered appearance of the animal’s coat.

Both receptors were observed in the sweat and sebaceous glands. The presence of ADIPOR1 and ADIPOR2 has already been demonstrated by some authors at the level of the sebaceous gland of the human skin [23,24], where ADIPOQ can stimulate the proliferative and secreting activity of sebocytes [23]. The lipid synthesis alteration of the sebaceous gland can influence the skin barrier function and contribute to the pathogenesis of skin inflammatory processes. No differences were observed by immunohistochemistry between obese and normal weight dogs in terms of number of cells of the skin-associated lymphoid tissue (SALT) evaluated by IHC, as reported in a previous paper [14]. This can be attributed to the small animal sample or to the lack of a direct effect of the ADIPOQ-ADIPOR axis on the acquired immune system, despite its known effect on the natural immune system, namely on macrophages that are shifted from an M1 to an M2 phenotype [58]. In atopic dermatitis, the proliferation and secreting activities of the sebaceous glands are reduced and associated with lower skin hydration [59]. Low plasma ADIPOQ levels have been associated with an increase in the manifestation of atopic dermatitis in mice [60]. 

Biological properties of ADIPOQ depend on both its plasmatic and peripheral concentration and the expression of specific tissue receptors. The physiological role of ADIPOQ receptors in mediating the ADIPOQ effects remain to be elucidated in dogs.

The decreased effect of adiponectin, in regard to obese dog skin, could essentially involve the receptor isoform 2. Indeed, in our study, ADIPOR2 appeared downregulated in obese dog skin while ADIPOR1 did not show significant differences. *ADIPOR2* mRNA expression, but not ADIPOR1, correlates with cutaneous expression and circulating levels of ADIPOQ. Bjursell et al. [61] observed that ADIPOR1 and ADIPOR2 are clearly involved in the energy metabolism of a mouse, but with opposing effects. Different ADIPOR1 and ADIPOR2 gene expression was described in brown fat and subcutaneous white fat of a fasted mouse [62], demonstrating that the two receptors behave differently following a stimulus. In agreement with our study, in humans and pigs, decreased expression of ADIPOR2, but no differences for ADIPOR1, were observed in visceral and subcutaneous adipose tissue in obese subjects compared to lean controls [63,64]. Patients affected by impaired glucose tolerance and type 2 diabetes showed reduced *ADIPOR2* mRNA expression in subcutaneous fat, whereas *ADIPOR1* mRNA was higher. Lord et al. [35] observed that TNFα interferes with ADIPOQ function by downregulation of *ADIPOR2,* but not of *ADIPOR1* mRNA levels in pig visceral fat tissues. It was hypothesized that *ADIPOR1* mRNA might be able to compensate for decreased expression of *ADIPOR2* in pig visceral fat and, thus, be able to mediate ADIPOQ effects in fat tissues. A compensation activity between the two receptors can also be hypothesized in dog skin. 

## 5. Conclusions

Obesity and associated diseases are a growing concern in pets. Obesity can negatively affect the physiology of the skin, promoting the manifestation of skin diseases, such as ulcerations, infections, delayed healing of wounds, and even skin cancer [65,66], although few studies have investigated this relationship in dogs. 

Our investigation showed that the expression of ADIPOQ and ADIPOR2 in dog skin is negatively correlated with obesity conditions, while ADIPOR1 did not show significant differences. Considering that serum ADIPOQ concentration and its skin expression were altered in obesity, these findings suggest that ADIPOQ’s effects on skin are at least in part regulated by the reduced expression of ADIPOR2. These data must be considered in the evaluation and treatment of diseases. The study confirms that ADIPOQ systems change in the dog’s skin in obesity conditions, leading to interesting questions about the actions of adipokines at the integumentary system level. The study may provide new perspectives in the field of dermatological research, even in pathological conditions, including the regenerative processes of wounds, endocrine dysfunctions, and the different types of alopecia that significantly affect the canine species.

## Figures and Tables

**Table 1:** Primers for *ADIPOQ*, *ADIPOR1*, and *ADIPOR2* housekeeping gene used for RT-qPCR quantification.

Gene	NCBI Seq. Ref.		Primers	bp
*ADIPOQ*	NM_001003070.1	F	TTCATCTGGAAGTGGGCGAC	107
		R	AAGGAAGCCCGTAAAGGTGG	
*ADIPOR1*	XM_843263.5	F	GCAGACAAGAGCAGGAGTGT	130
		R	AGCCATGAGGAAGAACCAGC	
*ADIPOR2*	NM_001024634.1	F	GGTCTCCCGGCTCTTCTCTA	145
		R	AATGCCCAGCACACAGATGA	
*ACTB* [14]	NM_001195845.2	F	CTTCCAGCCTTCCTTCCTGG	141
		R	CCAGGGTACATGGTGGTTCC	

**Table 2:** Serum biochemical analysis and serum ADIPOQ profile in obese and normal weight dogs.

Parameter	Unit	Reference Ranges	Normal Weight Group	Obese Group	*p*
Glucose	mg/dL	65–118	77.3 ± 10.14	82.6 ± 8.41	0.110
Urea	mg/dL	21–59	37.7 ± 7.56	41.5 ± 11.15	0.192
Creatinine	mg/dL	0.5–1.5	1.3 ± 0.14	1.4 ± 0.2	0.067
T-Chol	mg/dL	135–270	154.5 ± 30	151.2 ± 34.84	0.411
TG	mg/dL	20–112	38.9 ± 4.79	48.4 ± 15.99	0.052
ALT	UI/L	21–102	37.2 ± 10.72	39.3 ± 24.34	0.403
GGT	UI/L	1.2–6.4	3 ± 1.05	5.1 ± 2.85	0.025 *
ALP	UI/L	20–156	66.7 ± 35.1	70.6 ± 49.15	0.420
T-Bil	mg/dL	0.1–0.5	0.1 ± 0.02	0.3 ± 0.19	0.016 *
TP	g/dL	5.4–7.1	6.8 ± 0.42	7.1 ± 0.62	0.144
Alb	g/dL	2.6–3.3	3.4 ± 0.40	3.5 ± 0.27	0.163
α1-glob	g/dL	0.2–0.5	0.2 ± 0.02	0.2 ± 0.05	0.004 **
α2-glob	g/dL	0.3–1.1	0.9 ± 0.15	1.0 ± 0.13	0.087
β1-glob	g/dL	0.7–1.3	0.9 ± 0.35	0.8 ± 0.23	0.135
β2-glob	g/dL	0.6–1.4	0.8 ± 0.23	0.8 ± 0.15	0.323
γ-glob	g/dL	0.5–1.3	0.6 ± 0.14	0.7 ± 0.25	0.054
ADIPOQ	pg/mL	-	607.15 ± 220.2	183.05 ± 83.2	0.000 **

Data are expressed as mean ± standard deviation. T-Chol: total cholesterol; TG: total triglycerides; ALT: alanine aminotransferase; GGT: gamma-glutamyl transferase; ALP: alkaline phosphate; T-Bil: total bilirubin; TP: total proteins, Alb: albumins; α1-glob: α1-globulins; α2-glob: α2-globulins; β1-glob: β1-globulins; β2-glob: β2-globulins; γ-glob: γ-globulins. Reference ranges: Kaneko et al. [38]. * *p* < 0.05; ** *p* < 0.0001.

**Table 3:** Mean mRNA levels and standard deviation values (SD) of *ADIPOQ* and its receptors (*ADIPOR1*, *ADIPOR2*) in normal weight and obese skin dogs.

	Group	Nw	Ob	*p* * Nw vs. Ob
*ADIPOQ*	Mean	1.13	0.21	0.005
	SD	0.45	0.08	
*ADIPOR1*	Mean	4.83	42.16	0.068
	SD	3.71	31.40	
*ADIPOR2*	Mean	1.04	0.46	0.03
	SD	0.22	0.26	

RT-qPCR mRNA expressions for *ADIPOQ*, *ADIPOR1*, and *ADIPOR2* in skin collected in normal weight and obese dogs. Data are represented as the fold changes of mRNA expression in normal weight dogs to those in obese dogs, to an *ACTB* housekeeping gene. The relative abundances of target genes were calculated using the 2^−ΔΔ^Ct method. The means ± SD of *ADIPOQ*, *ADIPOR1*, and *ADIPOR2* mRNA expression levels for the three mRNA measurements were calculated for 10 animals/group. * Significantly different values were considered at *p* < 0.01 between normal weight and obese groups. *ADIPOQ*: adiponectin; *ADIPOR1*: adiponectin receptor 1; *ADIPOR2*: adiponectin receptor 2; Nw: normal weight group; Ob: obese group.

## Data Availability

The data presented in this study are available on request from the corresponding author.
